# *XPD* c.934G>A polymorphism of nucleotide excision repair pathway in outcome of head and neck squamous cell carcinoma patients treated with cisplatin chemoradiation

**DOI:** 10.18632/oncotarget.7668

**Published:** 2016-02-24

**Authors:** Leisa Lopes-Aguiar, Ericka Francislaine Dias Costa, Guilherme Augusto Silva Nogueira, Tathiane Regine Penna Lima, Marília Berlofa Visacri, Eder Carvalho Pincinato, Luciane Calonga, Fernanda Viviane Mariano, Albina Messias de Almeida Milani Altemani, João Maurício Carrasco Altemani, Cláudia Malheiros Coutinho-Camillo, Maria Almerinda Vieira Fernandes Ribeiro Alves, Patrícia Moriel, Celso Dario Ramos, Carlos Takahiro Chone, Carmen Silvia Passos Lima

**Affiliations:** ^1^ Department of Internal Medicine, Faculty of Medical Sciences, University of Campinas, Campinas, São Paulo, Brazil; ^2^ Department of Clinical Pathology, Faculty of Medical Sciences, University of Campinas, Campinas, São Paulo, Brazil; ^3^ Department of Ophthalmology and Otorhinolaryngology, Faculty of Medical Sciences, University of Campinas, Campinas, São Paulo, Brazil; ^4^ Department of Pathology, Faculty of Medical Sciences, University of Campinas, Campinas, São Paulo, Brazil; ^5^ Department of Radiology, Faculty of Medical Sciences, University of Campinas, Campinas, São Paulo, Brazil; ^6^ Department of Pathology, A.C. Camargo Cancer Center, São Paulo, São Paulo, Brazil

**Keywords:** head and neck squamous cell carcinoma, cisplatin, nucleotide excision repair pathway, single nucleotide polymorphisms, outcome

## Abstract

This study aimed to investigate the associations of *XPC* c.2815A>C, *XPD* c.934G>A and c.2251A>C, *XPF* c.2505T>C and *ERCC1* c.354C>T single nucleotide polymorphisms (SNPs) of nucleotide excision repair pathway in outcome of head and neck squamous cell carcinoma (HNSCC) patients treated with cisplatin (CDDP) chemoradiation. Patients with *XPC* c.2815AC or CC and *XPD* c.934GA or AA genotypes had 0.20 and 0.38 less chances of presenting moderate/severe ototoxicity and nausea, respectively. Patients with *XPD* c.934AA and c.2251AC or CC genotypes had 8.64, 12.29 and 3.55 more chances of achieving complete response (CR), consistent ototoxicity and nephrotoxicity, respectively. AA haplotype of *XPD* and ACT haplotype of *XPD* and *ERCC1* SNPs were associated with 9.30 and 3.41 more chances of achieving CR and consistent nephrotoxicity, respectively. At 24 months of follow-up, patients with *XPD* c.934AA genotype presented lower progression-free survival and overall survival in Kaplan-Meier estimates, and differences between groups remained the same in univariate Cox analysis. Patients with *XPD* c.934AA genotype had 2.13 and 2.04 more risks of presenting tumor progression and death than others in multivariate Cox analysis. Our data present preliminary evidence that *XPC* c.2815A>C, *XPD* c.934G>A and c.2251A>C, and *ERCC1* c.354C>T SNPs alter outcome of HNSCC patients treated with CDDP chemoradiation.

## INTRODUCTION

Head and neck squamous cell carcinoma (HNSCC) is the sixth most common human cancer, with a worldwide incidence of 600,000 new cases and approximately 350,000 deaths are attributed to tumor each year [1].

About two-thirds of HNSCC patients exhibit advanced stage disease at diagnosis [2], and cisplatin (CDDP) associated with radiotherapy (RT) has been used in their treatment [3]. RT induces DNA damage directly by action of photons and indirectly by liberation of free radicals [4]. CDDP develops adducts with cellular DNA and also releases free radicals [5]. In both cases, damaged cells are induced to apoptosis when not adequately repaired, particularly by nucleotide excision repair (NER) pathway [6].

The *xeroderma pigmentosum* (*XP*) genes, including complementation group C (*XPC*), D (*XPD*), F (*XPF*) and excision repair cross-complementation group1 (*ERCC1*), operate in NER pathway, and participate of recognition, demarcation and removal of DNA damage induced by CDDP and RT [7].

Variations in tumor sensitivity to CDDP [8–21], RT [22, 23] and CDDP associated with RT [24–26], as well as in side effects of therapeutic modalities [10, 14, 27, 28], have been attributed to distinct activities of proteins encoded by single nucleotide polymorphisms (SNPs) in genes involved in DNA repair through NER pathway.

The variant alleles of *XPC* c.2815A>C (p.Lys939Gln) (rs2228001), *XPD* c.934G>A (p.Asp312Asn) (rs1799793) and *XPD* c.2251A>C (p.Lys751Gln) (rs13181) SNPs determine activity of protein reduction, with consequent lower function in DNA repair capacity (DRC) [29, 30]. The variant alleles of *XPF* c.2505T>C (p.Ser835Ser) (rs1799801) and *ERCC1* c.354C>T (p.Asn118Asn) (rs11615) SNPs can be associated with a reduction of mRNA stability or processing, and lower DRC [31–33].

The *XPC* c.2815A>C [14–17], *XPD* c.934G>A and c.2251A>C [8–10, 13, 14, 19, 20, 24, 26, 28], *XPF* c.2505T>C [26], and *ERCC1* c.354C>T [10–12, 15, 16, 18, 21, 25, 26, 28] SNPs were associated with variable response rate (RR), toxicity, progression-free survival (PFS) and overall survival (OS) in patients with different tumors treated with CDDP-based chemotherapy with or without RT; however only few studies were conducted in HNSCC patients [24, 25].

In the present study, we investigated whether the above-mentioned SNPs alter the outcome of HNSCC patients treated with CDDP and RT.

## RESULTS

### Study population

Most of 90 patients enrolled in study were male and with a history of tobacco and alcohol consumption. About two-thirds of cases had tumor in pharynx and most of patients presented well or moderately differentiated tumor and tumor in advanced stages. Human papillomavirus (HPV) type 16 was negative in all analyzed cases (Table 1).

**Table 1:** Clinical characteristics and tumor aspects of head and neck squamous cell carcinoma patients

Variable	Median (range) or N (%)
**Age (years)**	56 (27-74)
**Gender**	
Male	83 (92.2)
Female	7 (7.8)
**Body mass index (kg/m^2^)**	19 (13-31)
**Tobacco consumption**	
Smokers	88 (97.8)
Non-smokers	2 (2.2)
**Alcohol consumption**	
Drinkers	83 (92.2)
Abstainers	7 (7.8)
**Tumor location**	
Oral cavity	12 (13.3)
Pharynx	55 (61.1)
Larynx	23 (25.6)
Histological grade*	
Well + moderately	60 (82.2)
Poorly + undifferentiated	13 (17.8)
**Tumor stage**	
I + II	6 (6.7)
III + IV	84 (93.3)
Human papillomavirus type 16*	
Positive	0 (0.0)
Negative	57 (100.0)

(N) number of patients. *The number of patients differed from the total quoted in the study (n= 90), because it was not possible to obtain consistent information about histological grade and human papillomavirus type 16 status in some cases.

All patients received RT with a total dose of 70 Gy and CDDP at initial dose of 80-100 mg/m^2^. Thirteen patients with consistent side effects after the first infusion of CDDP, received lower dose (50-75 mg/m^2^) of agent in following administrations. Sixty-eight patients (75.5%) received three infusions of CDDP and 22 patients (24.5%) received only two CDDP infusions due to renal or hematologic toxicities; the median cumulative dose of CDDP in patients was 265 mg (range: 100 to 616). Most of patients (97.7%) had medium or high adherence to antiemetics.

Partial response and stable disease were seen in near 80.0% of patients. About two-thirds and one-third of cases had moderate/severe nausea and vomiting, respectively, one-third to half of cases presented moderate/severe hematologic toxicities and half of cases had moderate/severe nephrotoxicity or ototoxicity (Table 2).

**Table 2:** Responses and toxicities to chemoradiotherapy of head and neck squamous cell carcinoma patients

Variable	Ideal or mild		Non-ideal, moderate or severe	
	Type of response or grade of toxicity	N (%)	Type of response or grade of toxicity	N (%)
**Response rate**	CR+PR	68 (93.2)	SD	5 (6.8)
	CR	15 (20.5)	PR+SD	58 (79.5)
**Gastrointestinal toxicities**				
Nausea	G0+G1	37 (42.0)	G2+G3	51 (58.0)
Vomiting	G0+G1	59 (67.0)	G2+G3+G4	29 (33.0)
**Hematologic toxicities**				
Anemia	G0+G1	37 (44.0)	G2+G3+G4	47 (56.0)
Leukopenia	G0+G1	47 (56.0)	G2+G3+G4	37 (44.0)
Neutropenia	G0+G1+G2	67 (79.8)	G3+G4	17 (20.2)
Lymphopenia	G0+G1+G2	42 (50.0)	G3+G4	42 (50.0)
Thrombocytopenia	G0	54 (64.3)	G1+G2+G3+G4	30 (35.7)
**Nephrotoxicity**	G0+G1	36 (52.2)	G2+G3+G4+G5	33 (47.8)
**Ototoxicity**	G0+G1	36 (51.4)	G2+G3+G4	34 (48.6)

(N) number of patients; (CR) complete response; (PR) partial response; (SD) stable disease; (G) grade of toxicity. The total number of patients differed from the total quoted in the study (n= 90), because it was not possible to obtain consistent information about response rate, nausea and vomiting, hematologic exams, glomerular filtration rate or audiometry test after chemoradiotherapy in some cases.

The mean ± standard deviation of urinary CDDP was 237.0 μg/mg ± 116.2.

The median follow-up time of 90 HNSCC patients enrolled in study was 18.6 months (range: 3.3-48.9). The estimated probabilities of 24-months PFS and OS were 37.6% and 42.4%, respectively. At the date of analysis, September 2015, 31 patients were alive, 7 of them with HNSCC and 24 without HNSCC and 59 patients died, 56 of them by the tumor effects and 3 by unrelated causes.

The linkage disequilibrium (LD) analysis revealed a LD between *XPD* c.934G>A and *XPD* c.2251A>C (D’= 64%), *XPD* c.934G>A and *ERCC1* c.354C>T (D’= 54%), and *XPD* c.2251A>C and *ERCC1* c.354C>T (D’= 51%) SNPs. From the theoretical eight possible *XPD* haplotypes for c.934G>A and c.2251A>C SNPs, four were found to be common (frequency > 1%: GA, GC, AA, AC). Only seven out of eighteen possible *XPD* and *ERCC1* haplotypes for c.934G>A, c.2251A>C and c.354C>T SNPs were found to be common (frequency > 1%: GAC, GCC, AAC, ACC, GAT, GCT, ACT). The common haplotypes of referred SNPs were included in further analysis.

### Polymorphisms, response rate and toxicity

**Figure 1:**
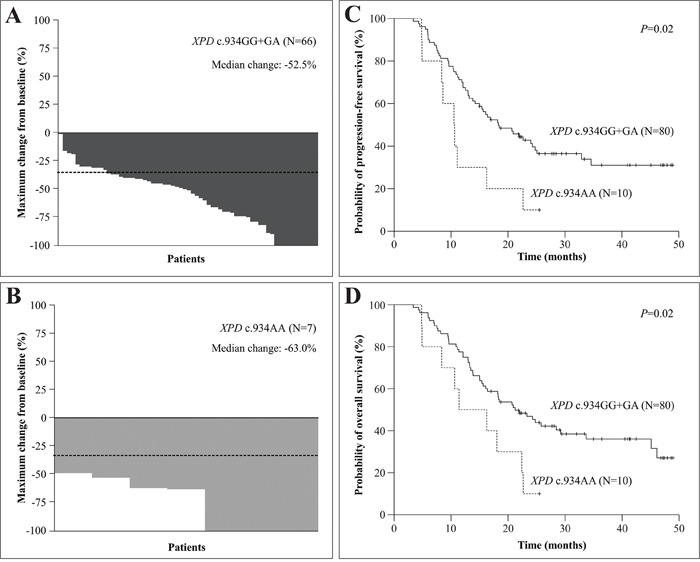
Characteristics of response to cisplatin-chemoradiotherapy and probability of progression-free and overall survival of head and neck squamous cell carcinoma (HNSCC) patients **Panels A** and **B**. show the waterfall plots indicate the maximum change from baseline in the sum of reference diameters of target lesion in with *XPD* c.934G>A genotypes. The dashed lines indicate a 30% reduction in the tumor burden in the target lesion, as defined by Response Evaluation Criteria in Solid Tumors version 1.1. **Panels C** and **D**. show Kaplan-Meier curve for progression-free and overall survival among HNSCC patients with XPD c.934G>A genotypes.

The frequencies of referred genotypes and haplotypes of HNSCC patients stratified by RR and toxicity to chemoradiotherapy are presented in Table 3. The *XPC* c.2815AC or CC genotypes were less common than AA genotype in patients with moderate/severe ototoxicity (40.4% *versus* 65.2%). Patients with AC or CC genotypes had 0.20 less chance of moderate/severe ototoxicity than others. The *XPD* c.934AA variant genotype was more frequent than GG or GA genotypes in patients with complete response (CR) after chemoradiotherapy (42.9% *versus* 18.2%). Carriers of variant genotype AA had 8.64 more chances of achieving CR than others. The maximum changes from baseline in the sum of reference diameters of target lesions in HNSCC patients with *XPD* c.934G>A SNP genotypes are presented in Figure 1A and 1B; patients with variant genotype had more median change than those with wild-type or heterozygous genotypes of *XPD* c.934G>A SNP (-63.0% *versus* -52.5%) of presenting response to chemoradiotherapy. The *XPD* c.934AA genotype was also more frequent than the GG or GA genotypes in patients with moderate/severe ototoxicity (85.7% *versus* 44.4%). Patients with AA genotype had 12.29 more chances of consistent ototoxicity than others. In contrast, the *XPD* c.934GA or AA genotypes were less common than the GG genotype in patients with moderate/severe nausea (48.8% *versus* 66.0%). Carriers of variant A allele had 0.38 less chance of moderate/severe nausea than those with the wild-type genotype. An excess of *XPD* c.2251AC or CC genotypes compared to the AA genotype were seen in patients with moderate/severe nephrotoxicity (62.2% *versus* 31.3%). Carriers of variant C allele had 3.55 more chances of consistent nephrotoxicity than others. The AA haplotype (variant allele of *XPD* c.934G>A and wild-type allele of *XPD* c.2251A>C) was more common in patients with CR than those with other common haplotypes (44.4% *versus* 19.0%). Individuals with AA haplotype had 9.30 more chances of achieving CR than others. The ACT haplotype (variant alleles of *XPD* c.934G>A, *XPD* c.2251A>C and *ERCC1* c.354C>T; respectively) was also more common in patients with moderate/severe nephrotoxicity than other haplotypes (70.0% *versus* 44.1%). Individuals with ACT haplotype had 3.41 more chances of consistent nephrotoxicity than others.

**Table 3:** Frequencies of *XPC* c.2815A>C, *XPD* c.934G>A, *XPD* c.2251A>C, *XPF* c.2505T>C and *ERCC1* c.354C>T single nucleotide polymorphisms genotypes and haplotypes of head and neck squamous cell carcinoma patients stratified by response rate and toxicity to chemoradiotherapy

Variable	Response rate				Nausea		Vomiting		Nephrotoxicity		Ototoxicity	
	CR+PR N (%)	SD N (%)	CR N (%)	PR+SD N (%)	G0+G1 N (%)	G2+G3 N (%)	G0+G1 N (%)	G2-G4 N (%)	G0+G1 N (%)	G2-G5 N (%)	G0+G1 N (%)	G2-G4 N (%)
***XPC* c.2815A>C**												
AA+AC	57 (93.4)	4 (6.6)	12 (19.7)	49 (80.3)	32 (42.1)	44 (57.9)	52 (68.4)	24 (31.6)	28 (48.3)	30 (51.7)	30 (51.7)	28 (48.3)
CC	11 (91.7)	1 (8.3)	3 (25.0)	9 (75.0)	5 (41.7)	7 (58.3)	7 (58.3)	5 (41.7)	8 (72.7)	3 (27.3)	6 (50.0)	6 (50.0)
*P*-value	0.80		0.34		0.79		0.46		0.07		0.65	
OR (95% CI)	0.72 (0.05-8.93)		2.20 (0.42-11.51)		1.19 (0.31-4.51)		1.63 (0.43-6.09)		0.23 (0.05-1.14)		1.36 (0.34-5.41)	
AA	24 (100.0)	0 (0.0)	7 (29.2)	17 (70.8)	11 (34.4)	21 (65.6)	20 (62.5)	12 (37.5)	12 (52.2)	11 (47.8)	8 (34.8)	**15 (65.2)**
AC+CC	44 (89.8)	5 (10.2)	8 (16.3)	41 (83.7)	26 (46.4)	30 (53.6)	39 (69.6)	17 (30.4)	24 (52.2)	22 (47.8)	28 (59.6)	**19 (40.4)**
*P*-value	0.99		0.32		0.27		0.65		0.79		**0.01**	
OR (95% CI)	NE		0.52 (0.14-1.88)		0.58 (0.22-1.52)		0.80 (0.30-2.10)		0.87 (0.30-2.49)		**0.20 (0.06-0.70)**	
***XPD* c.934G>A**												
GG+GA	61 (92.4)	5 (7.6)	**12 (18.2)**	54 (81.8)	32 (41.0)	46 (59.0)	53 (68.8)	25 (32.1)	33 (53.2)	29 (46.8)	35 (55.6)	**28 (44.4)**
AA	7 (100.0)	0 (0.0)	**3 (42.9)**	4 (57.1)	5 (50.0)	5 (50.0)	6 (60.0)	4 (40.0)	3 (42.9)	4 (57.1)	1 (14.3)	**6 (85.7)**
*P*-value	0.99		**0.04**		0.67		0.40		0.42		**0.03**	
OR (95% CI)	NE		**8.64 (1.04-71.76)**		0.73 (0.17-3.11)		1.84 (0.43-7.86)		2.00 (0.36-10.96)		**12.29 (1.19-126.04)**	
GG	36 (94.7)	2 (5.3)	8 (21.1)	30 (78.9)	16 (34.0)	**31 (66.0)**	33 (70.2)	14 (29.8)	21 (58.3)	15 (41.7)	17 (47.2)	19 (52.8)
GA+AA	32 (91.4)	3 (8.6)	7 (20.0)	28 (80.0)	21 (51.2)	**20 (48.8)**	26 (63.4)	15 (36.6)	15 (45.5)	18 (54.5)	19 (55.9)	15 (44.1)
*P*-value	0.66		0.77		**0.04**		0.58		0.23		0.54	
OR (95% CI)	0.65 (0.09-4.38)		0.83 (0.25-2.79)		**0.38 (0.14-0.98)**		1.29 (0.50-3.33)		1.83 (0.68-7.97)		0.73 (0.26-2.01)	
***XPD* c.2251A>C**												
AA+AC	60 (92.3)	5 (7.7)	13 (20.0)	52 (80.0)	35 (43.8)	45 (56.2)	55 (68.7)	25 (31.3)	33 (54.1)	28 (45.9)	31 (50.0)	31 (50.0)
CC	8 (100.0)	0 (0.0)	2 (25.0)	6 (75.0)	2 (25.0)	6 (75.0)	4 (50.0)	4 (50.0)	3 (37.5)	5 (62.5)	5 (62.5)	3 (37.5)
*P*-value	0.99		0.95		0.16		0.09		0.61		0.76	
OR (95% CI)	NE		1.05 (0.14-7.63)		3.50 (0.58-20.95)		4.11 (0.77-21.84)		1.51 (0.29-7.66)		0.77 (0.14-4.26)	
AA	32 (91.4)	3 (8.6)	7 (20.0)	28 (80.0)	17 (39.5)	26 (60.5)	27 (62.8)	16 (37.2)	22 (68.7)	**10 (31.3)**	16 (47.1)	18 (52.9)
AC+CC	36 (94.7)	2 (5.3)	8 (21.1)	30 (78.9)	20 (44.4)	25 (55.6)	32 (71.1)	13 (28.9)	14 (37.8)	**23 (62.2)**	20 (55.6)	16 (44.4)
*P*-value	0.73		0.81		0.46		0.40		**0.01**		0.75	
OR (95% CI)	1.39 (0.20-9.63)		1.15 (0.34-3.87)		0.70 (0.27-1.80)		0.65 (0.24-1.75)		**3.55 (1.27-9.87)**		0.85 (0.31-2.34)	
***XPF* c.2505T>C**												
TT+TC	60 (92.3)	5 (7.7)	14 (21.2)	52 (78.8)	33 (40.7)	48 (59.3)	53 (65.4)	28 (34.6)	33 (53.2)	29 (46.8)	34 (53.1)	30 (46.9)
CC	8 (100.0)	0 (0.0)	1 (14.3)	6 (85.7)	4 (57.1)	3 (42.9)	6 (85.7)	1 (14.3)	3 (42.9)	4 (57.1)	2 (33.3)	4 (66.7)
*P*-value	0.99		0.65		0.28		0.21		0.49		0.41	
OR (95% CI)	NE		0.58 (0.05-6.46)		0.39 (0.07-2.16)		0.23 (0.02-2.28)		1.77 (0.33-9.26)		2.17 (0.33-14.07)	
TT	25 (86.2)	4 (13.8)	7 (24.1)	22 (75.9)	16 (40.0)	24 (60.0)	28 (70.0)	12 (30.0)	20 (66.7)	10 (33.3)	13 (44.8)	16 (55.2)
TC+CC	43 (97.7)	1 (2.3)	8 (18.2)	36 (81.8)	21 (43.8)	27 (56.2)	31 (64.6)	17 (35.4)	16 (41.0)	23 (59.0)	23 (56.1)	18 (43.9)
*P*-value	0.11		0.53		0.69		0.56		0.07		0.30	
OR (95% CI)	7.17 (0.64-80.32)		0.67 (0.19-2.34)		0.83 (0.33-2.05)		1.32 (0.51-3.40)		2.56 (0.92-7.11)		0.58 (0.20-1.64)	
***ERCC1* c.354C>T**												
CC+CT	57 (93.4)	4 (6.6)	12 (19.7)	49 (80.3)	33 (45.2)	40 (54.8)	48 (65.8)	25 (34.2)	33 (57.9)	24 (42.1)	31 (52.5)	28 (47.5)
TT	11 (91.7)	1 (8.3)	3 (25.0)	9 (75.0)	4 (26.7)	11 (73.3)	11 (73.3)	4 (26.7)	3 (25.0)	9 (75.0)	5 (45.5)	6 (54.5)
*P*-value	0.67		0.79		0.15		0.61		0.06		0.47	
OR (95% CI)	0.58 (0.05-6.90)		1.23 (0.26-5.67)		2.50 (0.69-9.03)		0.71 (0.19-2.58)		4.00 (0.95-16.69)		1.63 (0.42-6.38)	
CC	20 (95.2)	1 (4.8)	6 (28.6)	15 (71.4)	11 (44.0)	14 (56.0)	16 (64.0)	9 (36.0)	12 (63.2)	7 (36.8)	12 (60.0)	8 (40.0)
CT+TT	48 (92.3)	4 (7.7)	9 (17.3)	43 (82.7)	26 (41.3)	37 (58.7)	43 (68.3)	20 (31.7)	24 (48.0)	26 (52.0)	24 (48.0)	26 (52.0)
*P*-value	0.87		0.54		0.88		0.47		0.13		0.68	
OR (95% CI)	0.82 (0.07-8.58)		0.66 (0.18-2.44)		1.07 (0.39-2.95)		0.68 (0.24-1.94)		2.43 (0.75-7.85)		1.27 (0.39-4.11)	
***XPD*+*XPD***												
AA	8 (88.9)	1 (11.1)	**4 (44.4)**	5 (55.6)	7 (58.3)	5 (41.7)	6 (50.0)	6 (50.0)	5 (71.4)	2 (28.6)	4 (44.4)	5 (55.6)
Other haplotypes	128 (93.4)	9 (6.6)	**26 (19.0)**	111 (81.0)	67 (40.9)	97 (59.1)	112 (68.3)	52 (31.7)	67 (51.1)	64 (48.9)	68 (51.9)	63 (48.1)
*P*-value	0.87		**0.01**		0.13		0.24		0.49		0.63	
OR (95% CI)	0.83 (0.08-7.98)		**9.30 (1.67-51.77)**		0.36 (0.09-1.38)		2.05 (0.60-7.00)		0.55 (0.10-3.03)		1.42 (0.33-6.11)	
***XPD*+*XPD*+*ERCC1***												
ACT	18 (94.7)	1 (5.3)	3 (15.8)	16 (84.2)	11 (47.8)	12 (52.2)	15 (65.2)	8 (34.8)	6 (30.0)	**14 (70.0)**	9 (50.0)	9 (50.0)
Other haplotypes	118 (92.9)	9 (7.1)	27 (21.3)	100 (78.7)	63 (41.2)	90 (58.8)	103 (67.3)	50 (32.7)	66 (55.9)	**52 (44.1)**	63 (51.6)	59 (48.4)
*P*-value	0.70		0.56		0.36		0.97		**0.02**		0.79	
OR (95% CI)	1.54 (0.16-14.26)		0.66 (0.16-2.63)		0.64 (0.24-1.66)		1.01 (0.38-2.70)		**3.41 (1.18-9.87)**		1.15 (0.39-3.42)	

Similar frequencies of genotypes, alone or combined, and haplotypes of corresponding SNPs were seen in patients stratified by hematologic toxicities and concentration of CDDP in urine (data not shown).

### Polymorphisms and survival analysis

At 24 months of follow-up, shorter PFS was observed in patients with advanced tumor stage (34.4% *versus* 83.3%, *P*= 0.03) and *XPD* c.934AA genotype (10.0% *versus* 41.2%, *P*= 0.02) (Figure 1C); a shorter OS was also observed in patients with advanced tumor stage (38.4% *versus* 100.0%, *P*= 0.02) and *XPD* c.934AA genotype (10.0% *versus* 46.9%, *P*= 0.02) (Figure 1D) at this time (Kaplan-Meier estimates).

Associations of clinical and tumor characteristics and referred genotypes with survival of HNSCC patients in Cox analyses are presented in Table 4. In univariate Cox analysis, a tendency for shorter PFS and shorter PFS were seen in patients with advanced tumor stage and in those with *XPD* c.934AA genotype, respectively. Only the *XPD* c.934AA genotype was associated with shorter PFS in multivariate Cox analysis; individuals with *XPD* c.934AA genotype had 2.13 more risks to present tumor progression than those with the remaining genotypes. In univariate Cox analysis, a shorter OS was seen in patients with advanced tumor stage and in those with *XPD* c.934AA genotype. Only the *XPD* c.934AA genotype was associated with shorter OS in multivariate Cox analysis; individuals with *XPD* c.934AA genotype had 2.04 more risks of death than those with the remaining genotypes.

**Table 4:** Association of clinical and tumor characteristics, *XPC* c.2815A>C, *XPD* c.934G>A, *XPD* c.2251A>C, *XPF* c.2505T>C and *ERCC1* c.354C>T single nucleotide polymorphisms genotypes with survival of head and neck squamous cell carcinoma patients treated with chemoradiotherapy in univariate Cox analysis

Variables	Progression-free survival			Overall survival		
	N with event/N total	*P* value	HR (95% CI)	N with event/N total	*P* value	HR (95% CI)
**Age (years)**						
≤ 56	32/46	0.40	1.24 (0.74-2.06)	29/46	0.79	1.08 (0.57-2.05)
> 56	28/44			30/44		
**Gender**						
Male	54/83	0.22	1.68 (0.72-3.93)	55/83	0.86	1.10 (0.34-3.60)
Female	6/7			4/7		
**Tobacco consumption**						
Smokers	58/88	0.12	3.05 (0.73-12.67)	58/88	0.72	1.43 (0.19-10.44)
Non-smokers	2/2			1/2		
**Alcohol consumption**						
Drinkers	57/83	0.30	1.83 (0.57-5.89)	57/83	0.21	3.52 (0.48-25.71)
Abstainers	3/7			2/7		
**Tumor location**						
Oral cavity/oropharynx	36/51	0.47	1.20 (0.71-2.03)	36/51	0.24	1.48 (0.76-2.91)
Hypopharynx/larynx	24/39			23/39		
**Histological grade**						
Well/moderately	39/60	0.28	1.48 (0.71-3.08)	38/60	0.44	1.32 (0.64-2.75)
Poorly/undifferentiated	9/13			9/13		
**Tumor stage**						
I + II	1/6	0.06	6.64 (0.91-48.05)*	**1/6**	**0.05**	7.08 (0.97-51.28)^*^
III + IV	59/84			**58/84**		
***XPC* c.2815A>C**						
AA+AC	51/77	0.90	1.04 (0.51-2.12)	51/77	0.47	1.31 (0.62-2.77)
CC	9/13			8/13		
AA	21/32	0.82	1.06 (0.62-1.80)	22/32	0.58	1.15 (0.68-1.96)
AC+CC	39/58			37/58		
***XPD* c.934G>A**						
GG+GA	**51/80**	**0.02**	2.33 (1.13-4.77)^**^*	**50/80**	**0.02**	2.24 (1.09-4.61)^***^*
AA	**9/10**			**9/10**		
GG	30 / 48	0.38	1.25 (0.75-2.07)	31/48	0.75	1.08 (0.65-1.81)
GA+AA	30/42			28/42		
***XPD* c.2251A>C**						
AA+AC	56/82	0.46	1.45 (0.52-4.02)	55/82	0.51	1.40 (0.50-3.88)
CC	4/8			4/8		
AA	26/44	0.19	1.40 (0.84-2.33)	28/44	0.71	1.10 (0.66-1.83)
AC+CC	34/46			31/46		
***XPF* c.2505T>C**						
TT+TC	56/83	0.75	1.17 (0.42-3.24)	55/83	0.85	1.10 (0.39-3.04)
CC	4/7			4/7		
TT	26/41	0.60	1.14 (0.68-1.90)	25/41	0.40	1.24 (0.74-2.10)
TC+CC	34/49			34/49		
***ERCC1* c.354C>T**						
CC+CT	52/74	0.11	1.92 (0.91-4.06)	51/74	0.15	1.72 (0.81-3.65)
TT	8/16			8/16		
CC	19/25	0.35	1.29 (0.74-2.22)	18/25	0.64	1.13 (0.65-1.98)
CT+TT	41/65			41/65		

(N) number of patients; (HR) hazard ratio; (CI) confidence interval. Significant differences between groups are presented in bold letters. In multivariate Cox analysis (adjusted by tumor stage and *XPD* c.934G>A polymorphism): *P= 0.07, HR: 6.15, 95% CI: 0.84-44.68; ^*^P= 0.06, HR: 6.59, 95% CI: 0.90-47.90; ^**^*P= 0.03, HR: 2.13, 95% CI: 1.04-4.38; ^***^*P= 0.05, HR: 2.04, 95% CI: 1.00-4.20.

## DISCUSSION

We initially found that clinical and tumor aspects [3, 22, 24, 34, 35], RR, toxicity to chemoradiation and short survival in advanced tumor stages [3, 34–36] in our sample were similar to those previously described in other parts of world. Therefore, they were adequate for analysis of new prognostic factors in disease. Low prevalence of HPV infection was seen in our cases, as previously reported [37, 38], suggesting that the major factors enrolled in tumor development were tobacco and alcohol consumption.

Secondly, we found that *XPC* c.2815AC or CC genotype was associated with reduced ototoxicity. The AA genotype was related to less hearing impairment in osteosarcoma patients treated with CDDP [14]. Our finding was not expected, since the wild-type A allele of *XPC* c.2815A>C SNP was previously associated with higher DRC [30], and possibly with protection against hearing loss. However, variant C and wild-type A alleles of *XPC* c.2815A>C were also associated with similar DRC [39]. Thus, additional studies are required to evaluate the bind of *XPC* c.2815A>C with ototoxicity in HNSCC patients treated with CDDP chemoradiation.

Third, as previously reported, we found that AA genotype of *XPD* c.934G>A SNP was associated with CR in HNSCC patients [24]. The *XPD* c.934GA or AA genotypes and AA genotype were associated with reduced manifestation of nausea and moderate/severe ototoxicity in our cases, respectively. Nephrotoxicity was also more common in our patients with the *XPD* c.2251AC or CC genotypes. A possible explanation for these associations is that variant A and C alleles of *XPD* c.934G>A and c.2251A>C SNPs determine lower DRC [29], which could induced more apoptosis in tumor cells and normal outer hairs and renal tubular cells in response to CDDP chemoradiation. The reduced DRC in patients with GA or AA genotypes of *XPD* c.934G>A SNP may also induce more apoptosis in epithelial enterochromaffin cells of intestine of treated patients, resulting in absence of serotonin release, and consequent lack of stimuli in chemoreceptor trigger zone and vomiting center. No associations of *XPD* c.934G>A and c.2251A>C SNPs with RR and toxicities were seen in non-small cell lung cancer [8, 10, 12, 15, 19, 20, 26], osteosarcoma [14] and ovarian cancer [28] patients treated with CDDP with or without RT. The divergent results may be caused by differences in sample sizes, tumor types, antiemetic therapies, hydration conditions, and doses of CDDP in our and previously reported studies.

Fourth, the AA haplotype of *XPD* c.934G>A and c.2251A>C SNPs was associated with increased chance of obtaining CR, and nephrotoxicity was predominately seen in patients with ACT haplotype of *XPD* c.934G>A, c.2251A>C and *ERCC1* c.354C>T SNPs, indicating that the SNPs in *XPD* and *ERCC1* genes may act together in DRC, with effects on clinical manifestation in those patients.

Finally, we found shorter PFS and OS in patients with *XPD* c.934AA variant genotype. Corroborating our findings, this genotype was previously reported with shorter OS in non-small cell lung cancer patients treated with platinum [9, 19]. In contrast, *XPD* c.934AA variant genotype was related with longer PFS and/or OS in HNSCC [24] and esophageal cancer [13] patients, and did not influence survival in non-small cell lung cancer [8, 10, 12, 15, 20, 26] and ovarian cancer [28] patients treated with platinum or CDDP with or without RT. The variant allele of *XPD* c.934G>A SNP determines lower function in DRC [29], which may induced high apoptosis in response to CDDP chemoradiation in tumor cells and high RR in HNSCC. It is well known that the *TP53* gene has a crucial role in induction of apoptosis [40]; however, the tobacco consumption was associated with increased risk of *TP53* mutations in previous analyzed HNSCC patients [41], and might have produced the same effect in our cases. Facing these descriptions, we hypothesized that HNSCC patients with *XPD* c.934AA variant genotype and non-functional TP53 protein could lead to decrease DRC induced by CDDP chemoradiation and consequent decreased apoptosis of tumor cells. This could constitute a possible reason for the initial sensitivity to chemoradiotherapy and further poor prognosis (PFS and OS) seen in our cases, as previously reported in small cell lung cancer patients [42]. The divergent results seen in previous studies and our study may be attributed to different sample sizes, treatment types, antiemetic therapies, hydration conditions and follow-up times.

In conclusion, our findings presented preliminary evidence that *XPC* c.2815A>C, *XPD* c.934G>A, *XPD* c.2251A>C and *ERCC1* c.354C>T SNPs alter clinical outcome of HNSCC patients treated with CDDP chemoradiation. We believe that in the near future, pharmacogenetic studies in HNSCC can contribute to personalize treatment and optimize patient outcome.

## PATIENTS AND METHODS

### Patients, treatment and clinical variables

This prospective study comprised HNSCC patients seen at diagnosis at the Clinical Oncology Service of General Hospital of University of Campinas between June 2011 and February 2014. All patients were selected to CDDP chemoradiation as definitive treatment due to locoregional unresectable tumor, refusal of surgery facing expected functional or anatomic sequels, or an organ preservation protocol. Exclusion criteria were refusing to participate in study, low Karnofsky performance scale score and renal dysfunction. The study was conducted according to the Declaration of Helsinki and was approved by the institutional review board guidelines (n° 274/2011).

The data relating to age, gender, body mass index, tobacco and alcohol consumption, hematologic and biochemistry exams, tumor location, histological grade and stage were obtained from patient charts. Subjects were classified as smokers or non-smokers and drinkers or abstainers as previously reported [43]. The tumor was diagnosed by standard criteria [44] and staged by the criteria of American Joint Committee of Cancer [45].

HPV testing consisted of P16 immunohistochemistry in tumor fragments embedded in paraffin. Staining was regarded as positive if it was strong and diffuse (> 80% of tumor cells) and it was regarded as negative if absent or focal [46]. Wide spectrum HPV *in situ* hybridization was reserved for P16-positive cases. Punctate hybridization signals localized to the tumor cell nuclei in either analysis defined an HPV-positive tumor [47].

Concurrent single daily fractionated radiation (2 Gy/day) during 35 days and intravenous CDDP at initial dose of 80-100 mg/m^2^ on days 1, 22 and 43 was administered to patients; patients with consistent side effects during treatment received CDDP at lower dose [3, 48]. RR to chemoradiotherapy was assessed using Response Evaluation Criteria in Solid Tumors (RECIST) guidelines version 1.1 [49].

As hydration and antiemetic protocols, the patients received intravenous 3,000 ml of saline 0.9%, 125 ml of 20% mannitol, ondansetron 24 mg and dexamethasone 20 mg before CDDP infusion, as well as intravenous 2,000 ml of saline 0.9% and oral dexamethasone 8 mg (every 12 hours) and metoclopramida 10 mg (every 6 hours) during three days after each CDDP infusion [50, 51]. The antiemetics adherence was classified as high or medium adherence or non-adherence [52].

Nausea, vomiting, hematologic toxicities, nephrotoxicity and ototoxicity were assessed using information of adverse effects, hematologic exams, ^51^Cr-EDTA glomerular filtration rate and audiometric tests performed before and after chemoradiotherapy. The toxicities were evaluated according to the National Cancer Institute (NCI) criteria version 4.0 [53], and the worst grade for each toxicity in each patient was included in analysis.

CDDP in urine of patients collected 0 to 48 hours after each dose of CDDP was measured by high-performance liquid chromatographic [54]. The final concentration of urinary CDDP was considered as the sum of all measurements obtained after each administration of agent.

Surgical tumor resection was offered to patients with good clinical condition and partial response or tumor relapse. Patients not amenable to resection and with progressive disease or relapse received intravenous methotrexate at dose of 40 mg/m^2^ once a week until best response, limiting toxicity or progression of disease [55]. The follow-up of patients was performed at 3-month intervals. The end of follow-up period was September 2015.

### DNA extraction and genotyping

The genotyping procedure was performed using genomic DNA obtained from peripheral blood of patients and involved a polymerase chain reaction followed by the enzymatic digestion, as reported for *XPC* c.2815A>C [56], *XPD* c.934G>A and *XPD* c.2251A>C [57], *XPF* c.2505T>C [33] and *ERCC1* c.354C>T [58] SNPs. Positive and negative controls were used in all genotyping reactions. The amount of 15% of genotype determinations was carried out twice in independent experiments with 100% of concordance.

### Statistical analysis

The pairwise LD was performed using the Haploview 4.2 software to ensure that the markers were appropriate for inclusion in the *XPD* and *ERCC1* haplotype estimates. The LD was measured by the disequilibrium coefficient (D’). The D’ values ≤ 1 indicate LD.

The differences between groups were analyzed by chi-square (*χ^2^*) or Fisher’s exact test. Logistic regression model served to obtain odds ratios values, adjusted for clinicopathological aspects with *P*-values ≤ 0.10, with 95% confidence intervals (95% CI), to assess associations between SNPs genotypes, RR, nausea, vomiting, hematologic toxicities, nephrotoxicity and ototoxicity. ANOVA served to obtain values, adjusted for clinicopathological aspects with *P*-values ≤ 0.10, in assessment of associations between SNPs genotypes and urinary CDDP. This variable was transformed into ranks to perform the comparative analysis, since it was not normally distributed.

PFS and OS were defined as time interval between the date of diagnosis and the date of progression or relapse of disease, and the date of death by any causes or last follow-up, respectively. Kaplan-Meier method was used to plot PFS and OS curves, and log-rank test was applied to compare the distribution between groups. Multivariate Cox regression served to estimate hazard ratios values, adjusted for variables with *P*-values ≤ 0.10, with 95% CI, with the purpose of to assess the associations between SNPs genotypes, PFS and OS.

For statistical tests, significance was two-sided and achieved when *P*-values were ≤ 0.05. All tests were done using the SPSS 21.0 software.
